# Editorial Note: Discussing blockchain applications in TED Talks: A fashion wave approach to understanding the blockchain phenomenon

**DOI:** 10.1371/journal.pone.0331143

**Published:** 2025-08-27

**Authors:** The PLOS One Editors

This article [1] was identified as one of a series of submissions for which we have concerns that the peer review did not meet the expected standards for *PLOS One*. We regret that the issues were not identified and addressed prior to the article’s publication.

This note is not intended to question the quality of the article or contributions of the authors to the review process, as the Editors have no concerns regarding the authors’ involvement in the peer review.
